# The differential activation of intracellular signaling pathways confers the permissiveness of embryonic stem cell derivation from different mouse strains

**DOI:** 10.1242/dev.112375

**Published:** 2015-02-01

**Authors:** Satoshi Ohtsuka, Hitoshi Niwa

**Affiliations:** 1 Laboratory for Pluripotent Stem Cell Studies, RIKEN Center for Developmental Biology (CDB), Minatojima-minamimachi 2-2-3, Chuo-Ku, Kobe 650-0047, Japan; 2 CREST (Core Research for Evolutional Science and Technology), Japan Science Technology Agency, Honcho 4-1-8, Kawaguchi, Saitama 332-0012, Japan; 3 Laboratory for Development and Regenerative Medicine, Kobe University Graduate School of Medicine, 7-5-1 Kusunokicho, Chuo-ku, Kobe 6500017, Japan

**Keywords:** LIF signaling, MAP kinase, Stat3, Embryonic stem cell, Signal responsiveness

## Abstract

The requirement of leukemia inhibitory factor (LIF) for the establishment and maintenance of mouse embryonic stem cells (ESCs) depends on the genetic background of the ESC origin. To reveal the molecular basis of the strain-dependent function of LIF, we compared the activation of the intracellular signaling pathways downstream of LIF in ESCs with different genetic backgrounds. We found that the JAK-Stat3 pathway was dominantly activated in ESCs derived from ‘permissive’ mouse strains (129Sv and C57BL6), whereas the MAP kinase pathway was hyperactivated in ESCs from ‘non-permissive’ strains (NOD, CBA and FVB). Artificial activation of Stat3 supported stable self-renewal of ESCs from non-permissive strains. These data suggest that the difference in the balance between the two intracellular signaling pathways underlies the differential response to LIF.

## Abstract (precis)

**Highlighted article**: Different mouse strains show distinct JAK-Stat and MAPK activity responses to LIF treatment, accounting for the varying ease of generating stem cells from these lines.

## INTRODUCTION

Mouse embryonic stem cells (ESCs) were first established in fetal calf serum (FCS)-containing medium with mouse embryonic fibroblasts (MEF) feeder cells (Evans and Kaufman, 1981; Martin, 1981). The cytokine leukemia inhibitory factor (LIF) was identified as the activator to support self-renewal (Smith, et al., 1988). Supplementation of LIF into FCS-containing medium (FCS/LIF) allowed stable self-renewal of ESCs derived from 129 strains without MEF (Nichols et al., 1990). Combination of MEF with FCS/LIF supported ESCs with other genetic backgrounds than 129, but most of these were unstable in long-term culture (Kawase et al., 1994). Serum-free culture containing inhibitors for glycogen synthase kinase 3 (GSK3) and mitogen-activated protein kinase (MAPK) (2i) provided greatly improved culture conditions for any mouse strain (Ying et al., 2008; Nichols et al., 2009). Combination of 2i with LIF (2iLIF) was more suitable than 2i alone (Kiyonari et al., 2010). The establishment of ESCs from different genetic backgrounds in 2iLIF allowed us to revisit the question why LIF is sufficient to support self-renewal of ESCs derived from limited strains. Here, we demonstrate how ESCs from various genetic backgrounds respond to the LIF signal by assessing the quantitative balance in the activation of the intracellular signaling pathways.

## RESULTS AND DISCUSSION

### Comparison of the self-renewing abilities of ESCs derived from different strains

**Fig. 1.**
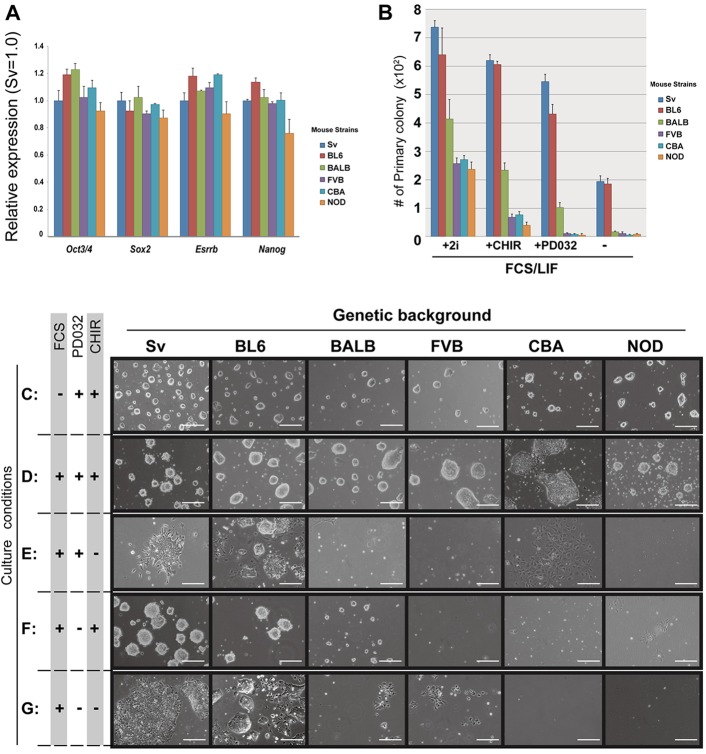
**Characterization of mouse ESC lines from various strains.** (A) qPCR analysis of expression levels of pluripotency-associated genes (*Oct3*/*4*, *Sox2*, *Esrrb*, *Nanog*) in ESCs derived from various strains, cultured in 2iLIF for five passages. (B) Primary colony formation ratio in FCS/LIF with inhibitors indicated. 129Sv ESCs in FCS/LIF plus 2i was used as control and set at 100%. (C-G) Primary colony formation of self-renewing ESCs derived from different strains in various culture conditions indicated. All ESCs were maintained on gelatin-coated dishes for 7 days at clonal density. Scale bars: 200 µm.

Previous reports indicated that there are two types of mouse strains: strains permissive for the establishment of ESCs in FCS/LIF or FCS/LIF/MEF (129Sv, C57BL6 and BALB/c), and non-permissive strains (NOD, CBA and FVB) (Kawase et al., 1994; Brook et al., 2003; Nagafuchi et al., 1999; Cinelli et al., 2008). We established three male ESCs of each type from these six strains using 2iLIF with MEF. These ESCs continued self-renewal, with maintaining expression of pluripotency-associated genes at comparable levels (Fig. 1A) and compact colony morphologies (Fig. 1C) in 2iLIF. The ability to produce germline chimeras was confirmed in ESCs derived from 129Sv and NOD (supplementary material Fig. S1).

We then tested their characteristics in other culture conditions. ESCs were seeded in 2iLIF, followed by incubation for 24 h. Then, the medium was changed to either 2iLIF or FCS/LIF with or without inhibitors (CHIR for GSK3, PD032 for MAPK or both). After culturing for 6 days, primary colony formation was evaluated by counting colony numbers (Fig. 1B) and by assessing colony morphologies (Fig. 1C-G). The efficiency of primary colony formation was significantly reduced upon removal of one of the inhibitors (Fig. 1B). In the presence of 2i, all ESCs formed stem cell colonies, even in FCS/LIF (Fig. 1C,D). However, in FCS/LIF, ESCs derived from 129Sv and C57BL6 formed stem-cell colonies at a much higher rate than ESCs derived from the other strains (Fig. 1G). Addition of either CHIR or PD032 to FCS/LIF (Fig. 1E,F) was insufficient to support stem cell colony formation of FVB-, CBA- and NOD-ESCs, although BALB/c-ESCs formed small, compact colonies. Addition of a higher dose of LIF (10^4^ units/ml) to FCS/LIF also failed to support stem cell colony formation in NOD-ESCs (supplementary material Fig. S2). These data indicate that FCS/LIF is insufficient to support self-renewal of BALB/c-, FVB-, CBA- and NOD-ESCs. BALB/c-ESCs have been previously referred to as permissive for derivation of ESCs in FCS/LIF with MEF (Baharvand and Matthaei, 2004); however, their characteristics were similar to those of non-permissive strains previously categorized (FVB, CBA and NOD) in FCS/LIF without MEF, even though the phenotypes with single inhibitors in FCS/LIF were intermediate (Fig. 1B,E,F). Therefore, hereafter we categorized 129Sv and C57BL6 as permissive, FVB, CBA and NOD as non-permissive strains, and BALB/c as intermediate strain.

### Differential activation of intracellular signaling pathways by LIF in strain-dependent manner

**Fig. 2.**
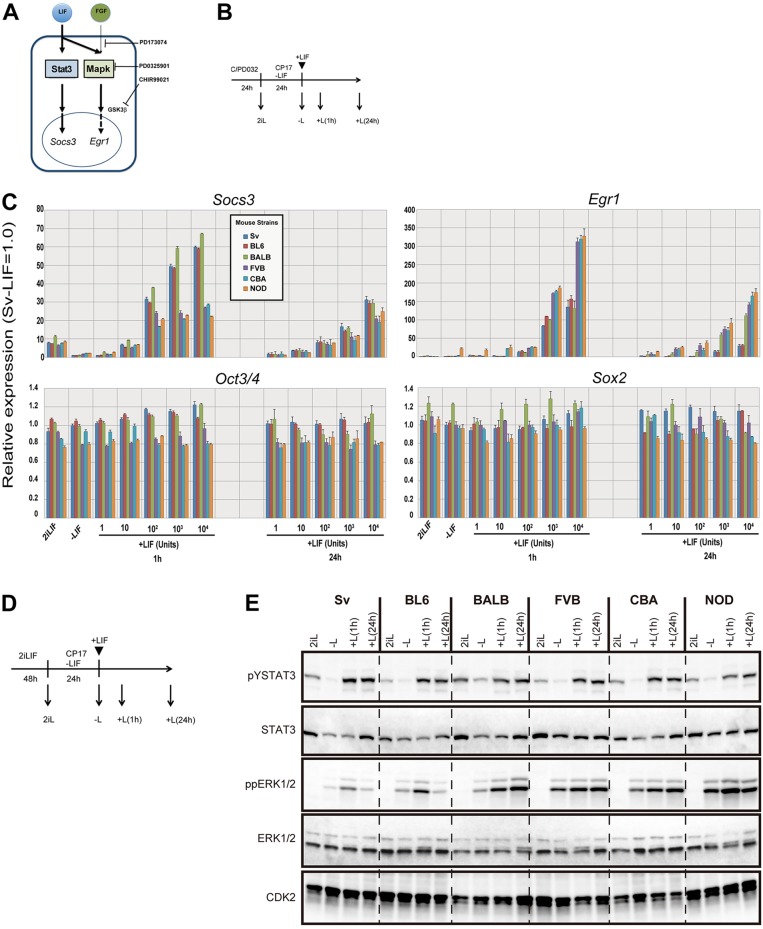
**Differential activation of intracellular signaling pathways by LIF in ESCs from different strains.** (A) Schematic of Jak-Stat3 and MAPK pathways under influence of the LIF signal. Targets of the specific inhibitors are indicated. (B) Experimental outline for analyzing LIF responsiveness in ESCs. See Materials and Methods for details. 2iLIF, −LIF and +LIF indicate the time points of RNA sampling for qPCR analyses. (C) qPCR analysis of expression of *Socs3*, *Egr1*, *Oct3/4* and *Sox2* in ESCs at each time point of the LIF responsive assay shown in B. The expression levels of the genes in 129Sv ESCs at −LIF were set at 1.0, and relative expression units (REUs) obtained by biological triplicates are shown; error bars indicate s.d. (D) Experimental outline for analyzing the phosphorylation state in different ESC lines. (E) Western blot analysis of different ESC lines after LIF stimulation. ESCs were plated into 2iLIF and cultured for 24 h (2iL).

We then tested the effect of the LIF signal on activation of the Jak-Stat3 and MAPK pathways, the positive and negative signals to promote self-renewal, respectively (Niwa et al., 2009). ESCs were cultured in 2iLIF for 24 h, followed by culture in N2B27 with CHIR and FGF receptor inhibitor (PD173074: PD17) to minimize the effect of the FGF signal on the MAPK pathway (Burdon et al., 1999; Kunath et al., 2007). Then, LIF at various concentrations was added, and gene expression profiles were analyzed after 1 h and 24 h. Expression levels of *Socs3* regulated by the JAK-Stat3 pathway (Endo et al., 1997) were comparable between all ESCs in 2iLIF and were similarly downregulated by withdrawal of LIF for 24 h. *Socs3* expression was activated by LIF in all ESCs, but its levels at 1 h showed a strain-dependent difference. Activation of *Socs3* showed dose dependency up to 10^4^ units/ml of LIF to 60 relative expression units (REUs) in permissive and intermediate strain-derived ESCs (pm-ESCs and im-ESCs, respectively) (Fig. 2C), whereas it was saturated with 10^2^ unit/ml of LIF around 20 REUs in non-permissive strain-derived ESCs (npm-ESCs). The opposite pattern was observed for activation of *Egr1*, a target of the MAPK pathway (Kawai-Kowase et al., 1999) (Fig. 2C). However, at 24 h, the difference in *Socs3* expression levels became moderate, whereas the difference in *Egr1* expression levels became more visible between pm-ESCs and npm-ESCs. Interestingly, expression levels of *Egr1* in im-ESCs were comparable to those in pm-ESCs at 1 h but became as high as those in npm-ESCs at 24 h. Expression levels of *Oct3/4* and *Sox2* were unchanged during culture, thus confirming their pluripotent state (Fig. 2C). We also examined the LIF responsiveness of these ESCs in serum-free culture with BMP4 (Ying et al., 2003) and obtained similar results (supplementary material Fig. S3). These observations were confirmed at protein level by monitoring phosphorylation of Stat3 and extracellular signal-related kinase (Erk) 1/2. Upon LIF stimulation, as shown in Fig. 2D, levels of phosphorylated Stat3 were increased in all ESCs, but the levels in pm-ESCs and im-ESCs were slightly higher than those in npm-ESCs (Fig. 2E). Levels of phosphorylated Erk1/2 were also increased in all ESCs after addition of LIF. Interestingly, at a short time period (1 h), there was no difference in levels of phosphorylated Erk1/2 among all ESCs. However, at the later period of 24 h, the difference became more distinct; levels of phosphorylated Erk1/2 decreased to very low levels in pm-ESCs and remained at high levels in npm-ESCs and im-ESCs (Fig. 2E). These data are consistent with the differential transcriptional activation of *Socs3* and *Egr1*, suggesting a quantitative difference in the activation of the Jak-Stat3 and MAPK pathways in pm-, im- and npm-ESCs. It has been reported that CBA- and FVB-ESCs were able to self-renew in serum-free culture with CHIR or PD032 but not in FCS/LIF (Wray et al*.*, 2010), which might due to hyperactivation of the MAPK pathway by FCS containing high FGF2 activity (Cushing et al*.*, 2008).

### Differential expression of signal transducers in ESCs from different strains

**Fig. 3.**
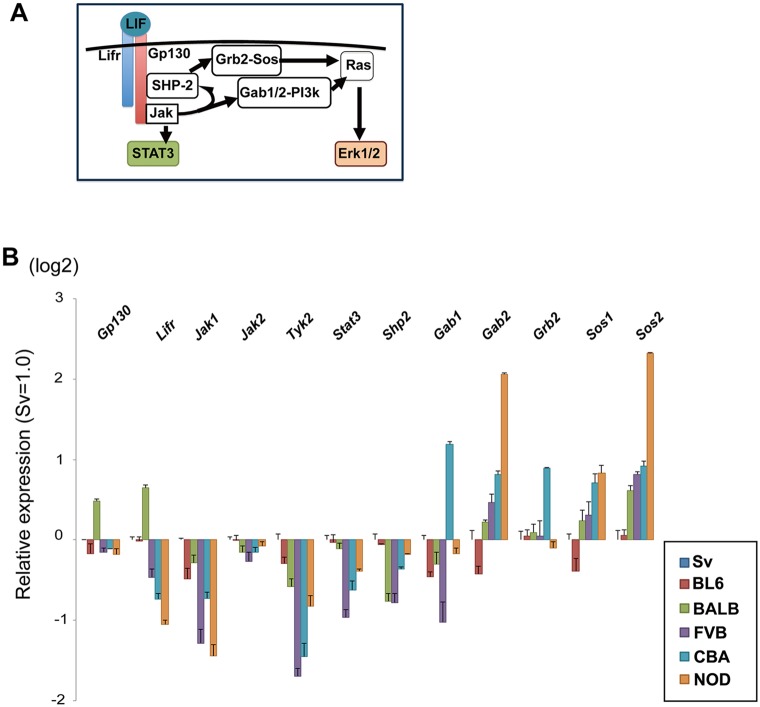
**Expression levels of LIF signaling components in ESCs from different strains.** (A) Schematic of the components of the Jak-Stat3 and MAPK pathways downstream of LIF in ESCs. (B) Relative expression levels of the LIF signaling components quantified by qPCR. Three independent ESC lines derived from each strain were cultured in 2iLIF and analyzed. The average REUs of the biological triplicates for each of the three ESC lines of the same genetic background (*n*=9) are shown; error bars indicate s.d. Gene expression levels in 129Sv ESCs were set at 1.0.

Next, we assessed expression levels of the components of the LIF signal transduction pathways summarized in Fig. 3A by quantitative PCR (qPCR) in ESCs cultured in 2iLIF. As shown in Fig. 3B, we found that the expression levels of the components of the Jak-Stat3 pathway were lower in npm-ESCs than in pm-ESCs. It has been reported that expression of *Lifr*, *Jak1* and *Stat3* is regulated by positive feedback via Stat3 (Bromberg et al., 1999). Therefore, their lower expression levels might be simply due to weaker activation of the Jak-Stat3 pathway by LIF in these strains. By contrast, the components of the Shp2-Ras-MAPK pathway, especially *Gab2* and *Sos2*, were expressed at higher levels in npm-ESCs than in pm-ESCs. As ESCs were maintained in 2iLIF in these experiments to repress Erk1/2 to undetectable levels by western blot (Fig. 2E), the difference could reflect genetic differences.

### Artificial activation of Stat3 supports self-renewal of npm-ESCs

**Fig. 4.**
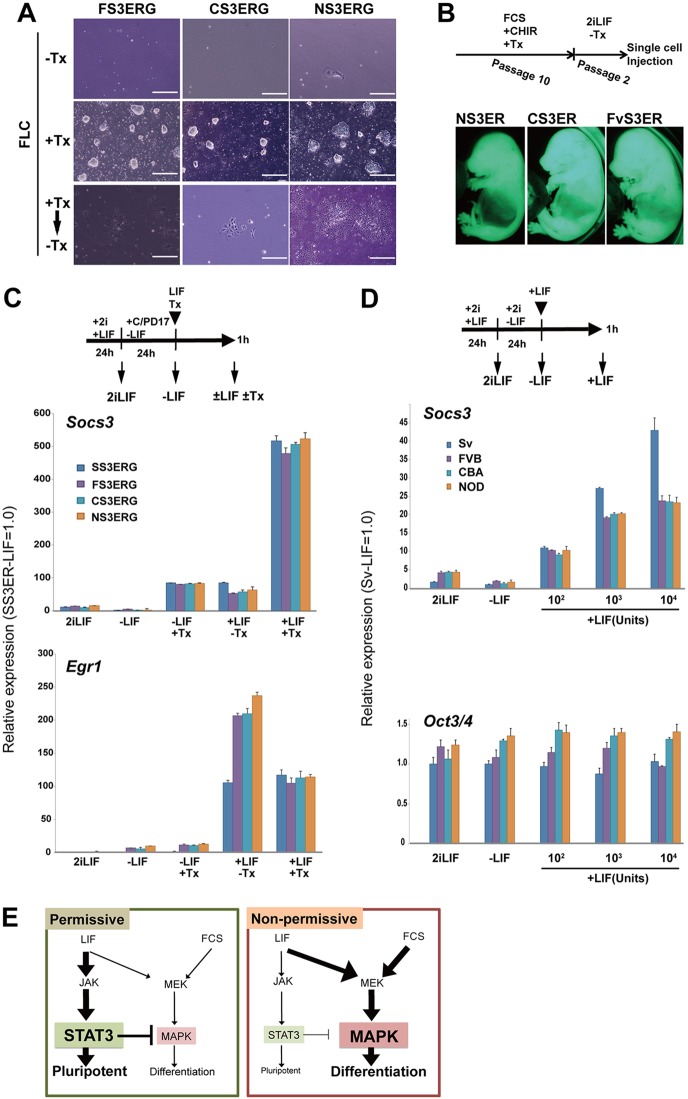
**Increased STAT3 activity supports self-renewal of npm-ESCs.** (A) Colony morphologies after increased Stat3 activity. FVB-, CBA- and NOD-derived ESCs carrying the Stat3ER transgene (FS3ERG, CS3ERG and NS3ERG, respectively) were maintained in FCS/LIF supplemented by CHIR (FLC) without Tx (−Tx), with Tx (+Tx) or after withdrawal of Tx (+Tx→−Tx). (B) Chimera assay to confirm pluripotency of the ESCs shown in A (+Tx). ESCs with Tx at passage 10 were maintained in 2iLIF without Tx for two passages. Then, a single ESC was injected into the blastocyst and the chimera embryos were confirmed at 13.5 dpc. (C) Effect of increased Stat3 activity on MAPK activity upon LIF stimulation, as evaluated by qPCR analysis. ESCs carrying Stat3ER were cultured in 2iLIF for 24 h (2iLIF), LIF was then depleted for 24 h in the presence of CHIR and PD17 (−LIF). Cells were stimulated with a combination of 10^3^ U/ml of LIF and Tx for 1 h and were analyzed by qPCR. SS3ERG is a 129Sv-derived ESC line carrying Stat3ER. (D) Effect of inhibition of MAPK on the Jak-Stat3 pathway in ESCs. ESCs were treated as in C, except for LIF depletion, which was performed in the presence of 2i (−LIF). Then, cells were stimulated with various doses of LIF (10^2^, 10^3^ and 10^4^ U/ml). (E) Proposed LIF signaling cascade in pm-and npm-ESCs.

The data shown above suggest that the disadvantage in activation of the Jak-Stat3 pathway could be the basis of npm-ESCs for self-renewal in FCS/LIF. To confirm this hypothesis, we tested the ability of artificial activation of Stat3 with a hormone-inducible form of Stat3 (Stat3ER) (Matsuda et al., 1999) to support self-renewal of npm-ESCs. Without activation of Stat3ER by 4-hydroxytamoxifen (Tx), transgenic npm-ESCs could not continue self-renewal as parental ESCs in FCS/LIF (Fig. 4A, top panels). With Tx, however, they continued self-renewal in FCS/LIF as pm-ESCs if CHIR is supplied (Fig. 4A, middle panels). These ESCs ceased self-renewal after withdrawal of either Tx or LIF (Fig. 4A, bottom panels). These npm-ESCs stably self-renewed in FCS/LIF with Tx and CHIR for a long time (ten passages), while keeping the ability to contribute to chimeric embryos (Fig. 4B).

We then tested the balance of the signaling pathways in these npm-ESCs carrying Stat3ER. They retained the poor response of *Socs3* to LIF without Tx. The activity of Stat3ER was almost similar among these transgenic ESC lines, as their expression levels of *Socs3* with Tx without LIF were comparable (Fig. 4C). With Tx and LIF, expression levels of *Socs3* were fivefold higher than those with either Tx or LIF (Fig. 4C), suggesting their high degree of synergy. These ESC lines also kept the hyperactivation of the MAPK pathway by LIF, as expression levels of *Egr1* were higher than those in 129Sv-derived ESCs (Fig. 4C). Interestingly, *Egr1* was repressed by activation of Stat3ER with Tx in these npm-ESCs (Fig. 4C). Therefore, the forced activation of Stat3 activity triggered activation of the canonical pathway and repression of the MAPK pathway. The combinatorial action might confer stable self-renewal on npm-ESCs in FCS/LIF.

Next, we tested the effect of activation of the MAPK pathway on the activity of the Jak-Stat3 pathway. Indeed, expression levels of *Socs3* in npm-ESCs remained low (Fig. 4D) in the presence of the MAPK inhibitor, and at comparable levels as with hyperactive MAPK (Fig. 2C). This indicates that the MAPK pathway has no effect on the activity of the Jak-Stat3 pathway under the LIF signal.

It has been reported that induced pluripotent stem cells (iPSCs) derived from NOD mice by conventional Yamanaka factors (Oct3/4, Sox2, Klf4 and Myc) require continuous expression of exogenous *Klf4* or *Myc* for their self-renewal in FCS/LIF (Hanna et al., 2009). Interestingly, both *Klf4* and *Myc* are known targets of the Jak-Stat3 pathway (Niwa et al., 2009; Cartwright et al., 2005). Thus, the disadvantage of the weak activation of the Jak-Stat3 pathway in npm-ESCs could be translated into the low levels of transcriptional activation of particular target genes.

### LIF responsiveness of rat ESCs is similar to mouse npm-ESCs

**Fig. 5.**
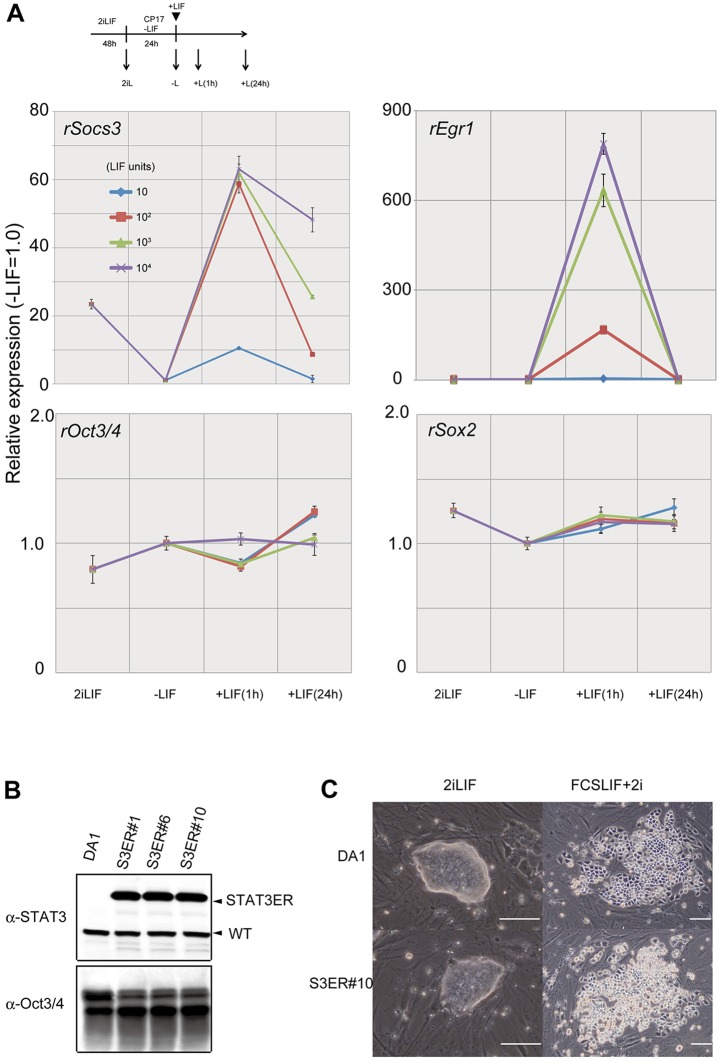
**LIF responsiveness of rat ESCs.** (A) Activation of *Socs3* and *Egr1* in rat ESCs with different concentration of LIF. Rat ESCs cultured in 2iLIF with MEF for 48 h were maintained with CHIR and PD17, followed by exposure to different concentrations of LIF. Expression levels of *Socs3*, *Egr1*, *Oct3/4* and *Sox2* were indicated as relative expression levels (with the expression levels of −LIF set at 1.0); error bars indicate s.e.m. (B) Expression of Stat3ER in rat ESCs. Protein expression in three independent rat ESC lines was confirmed by western blot. Oct3/4 was used as a control for proper loading of the samples. (C) Morphology of rat ESCs carrying Stat3ER cultured with Tx on MEF for 7 days in either 2iLIF (2iLIF) or FCS/LIF supplemented with 2i (FCSLIF+2i). Scale bars: 200 µm.

As rat ESCs can be established using 2iLIF culture (Buehr et al., 2008; Li et al., 2008; Isotani et al., 2011) but not with FCS/LIF (Buehr et al., 2003), they might have similar characteristics to those of mouse npm-ESCs. When we evaluated the LIF responsiveness of rat ESCs on expression of *Socs3* and *Egr1*, we found a similar pattern to that of mouse npm-ESCs. *Socs3* expression was saturated at a low concentration of LIF (10^2^ U/ml), whereas *Egr1* expression increased in a dose-dependent manner (Fig. 5A). However, three rat ESC lines with proper expression of the Stat3ER protein (Fig. 5B) underwent cell death rather than self-renewal with Tx and LIF, and even with 2i, which was also observed in wild-type rat ESCs (Fig. 5C). This suggested that rat ESCs are similar, but not identical, to mouse npm-ESCs in their character of LIF responsiveness.

Previous trials suggested that LIF is insufficient to establish and maintain ESCs from non-rodent species (Thomson et al., 1998; Honda et al., 2009; Sumi et al., 2004). As we found for rat ESCs, there might be additional factor(s) in ESCs from other species than mice that prevent proper LIF responsiveness in addition to that of mouse npm-ESCs. Further analysis of the molecular basis that determines the differential LIF responsiveness among mouse strains would contribute to understanding the difference between the species. This would provide a technique to establish naïve pluripotent stem cells from various species.

## MATERIALS AND METHODS

### Mice

Mouse strains employed to establish ESCs s were purchased from the following companies: 129^+*Ter*^/SvJcl, NOD/ShiJcl and FVB/NJcl (all from CLEA Japan), C57BL/6J and BALB/c (Charles River), and CBA/Ca (Harlan Laboratories).

### Animals ethics statement

All animal experiments conformed to the Guidelines for the Care and Use of Laboratory Animals and were approved by the Institutional Committee for Laboratory Animal Experiment (RIKEN Kobe Institute, Japan).

### Derivation and maintenance of ESCs

The FCS/LIF medium consists of Glasgow minimal essential medium (GMEM; Sigma) supplemented with 10% fetal calf serum (FCS), 1 mM sodium pyruvate, 10^−4^ M 2-mercaptoethanol, 1× non-essential amino acids and 1000 U/ml of LIF. For 2iLIF medium, N2B27 medium (Stem Cell Science) was supplemented with 100 U/ml of LIF, 3 µM CHIR99021 (Stemgent) and 1 µM PD0325901 (Stemgent).

Derivation and maintenance of ESCs were performed as described previously (Nichols et al., 2009). For LIF-responsive analysis, ESCs were plated at a density of 10^5^ cells/six-well dish in 2iLIF for 24 h. Then, cells were washed three times with N2B27 and kept for a further 24 h in N2B27 with 3 µM CHIR99021 and 100 nM PD173074 (C/PD17), followed by addition of LIF at the concentrations indicated. For the self-renewal assay, ESCs were plated at a clonal density (10^3^ cells per six-well dish) in 2iLIF for the first 24 h, cells were then washed with FCS/LIF three times, followed by culture in FCS/LIF with or without inhibitors as indicated in Fig. 1C-G for 7 days.

DA1Osb rat ESCs were maintained as described previously (Isotani et al., 2011).

### Analysis of ESCs carrying the Stat3ER and eGFP transgene

ESCs were transfected with pPB-CAG-Stat3ER-IP, pPBCAG-eGFP-IZ and pCAGGS-PBase, followed by culture with puromycin and zeocin with CHIR99021. The pools of transfected colonies were passaged in FCS/LIF with Tx and CHIR for ten passages (∼1 month). These cells were then cultured in 2iLIF without Tx for two passages and subjected to a chimera-formation assay by single ESC injection into blastocysts, as reported previously (Ohtsuka et al*.*, 2012).

### qPCR

qPCR was performed as described (Ohtsuka et al., 2012), with the primer sequences shown in supplementary material Table S1. To qualify the levels of transcripts, cDNAs were synthesized from 1 µg total RNA using ReverTra Ace (Toyobo) and evaluated by qPCR using a Bio-Rad CFX384 real-time system. All samples were tested in triplicate and the results of each were normalized relative to *Gapdh* expression.

## Supplementary Material

Supplementary Material
