# Supplementary material for: The differential activation of intracellular signaling pathways confers the permissiveness of embryonic stem cell derivation from different mouse strains
Source: Development. 2015 Feb 1;142(3):431–7. doi: 10.1242/dev.112375 (PMC4302992; doi:10.1242/dev.112375)
Supplement: Supplementary Material [file supp_142_3_431__index.html]

Supplementary Material 

# The differential activation of intracellular signaling pathways confers the permissiveness of embryonic stem cell derivation from different mouse strains

## DEV112375 Supplementary Material

**Files in this Data Supplement:**

- Supplementary Material
