# Supplementary material for: The differential activation of intracellular signaling pathways confers the permissiveness of embryonic stem cell derivation from different mouse strains
Source: Development. 2015 Feb 1;142(3):431–7. doi: 10.1242/dev.112375 (PMC4302992; doi:10.1242/dev.112375)
Supplement: Supplementary Material [file supp_dev.112375_DEV112375supp.pdf]

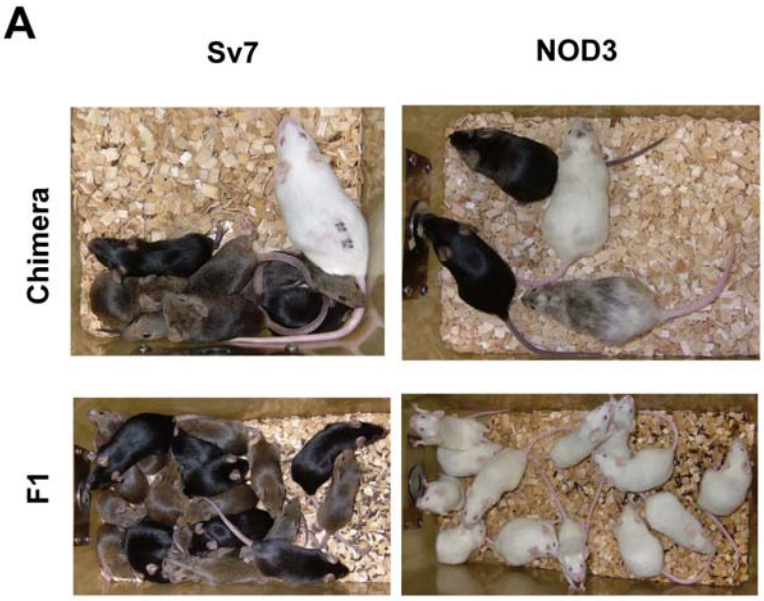

B

Cell line	# of germline transmission	# of chimera	# of offsprings	# of Injected
Sv3	10	12<br>10 males	15	80
Sv7	5	6<br>5 males	9	80
N3	2	2<br>2 males	8	80
N4	3	4<br>3 males	13	80

Supplemental Figure S1. Germ-lime competency of newly established ES cells from 129Sv and NOD.

ES cell lines from 129Sv and NOD strains we established were examined for their abilities to give rise to germline chimeras. A) Chimeric offsprings of injected 129Sv

(Sv7) and NOD (NOD3) ESCs. Single ESC was injected into B6 derived blastocyst individually and the embryos were transferred into the uterus of pseudo-pregnant CD1. At the day of birth, offspring were delivered by Caesarean section. Chimeric pups were distinguished by the chimeric coat color (129SV: agouti, NOD: albino, B6: black). B) Evaluation of germ line transmission ability through F1 production. All male chimeras obtained were mated with females (B6 for 129Sv chimeras, NOD for NOD-derived chimeras) and the germ line transmission ability was confirmed by the coat color of F1 offsprings.

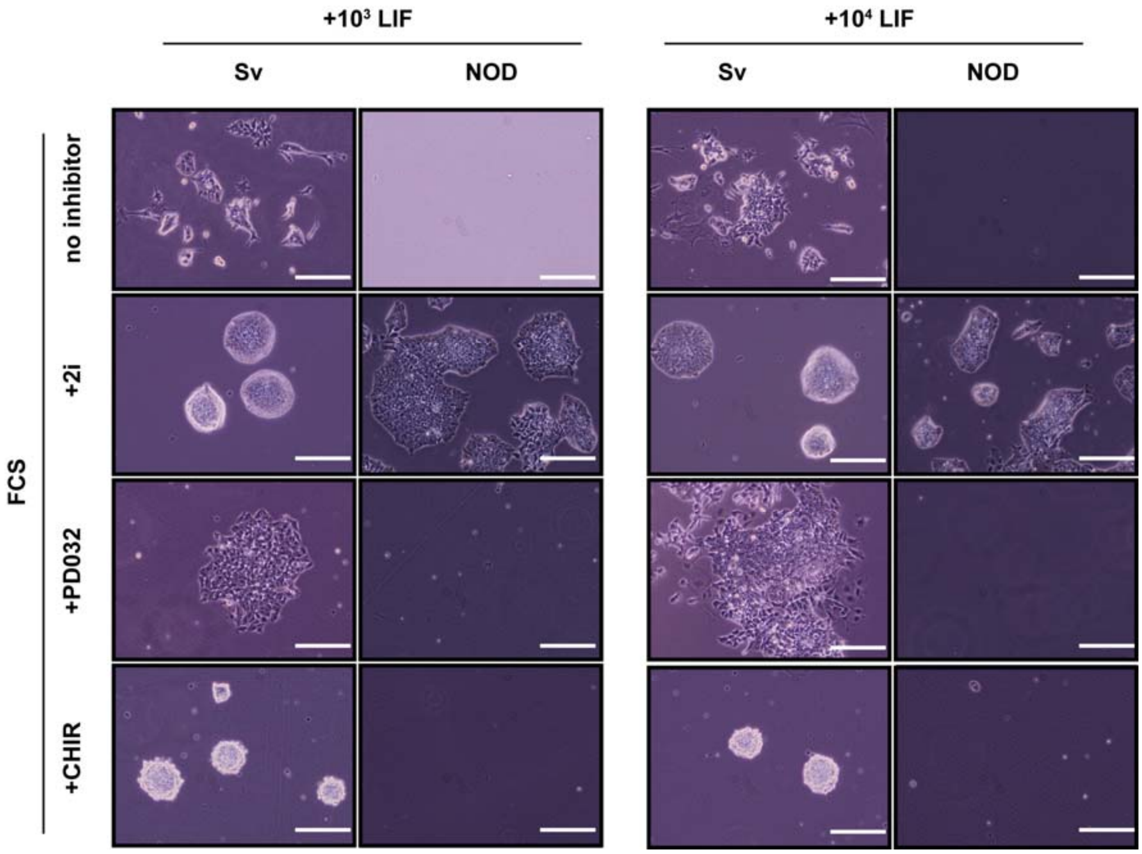

Supplemental Figure S2. The effect of LIF at high dose.

ES cells were maintained in FCS/LIF medium for 2 passages with either 10^3 or 10^4 units of LIF in combination of inhibitors as indicated. 129Sv-derived ES cells were self-renewing in all contexts (no inhibitors, +2i, +PD032 or +CHIR). However, NOD-derived ES cells were able to self-renew only in the presence of 2 inhibitors (+2i), but not in other conditions (no inhibitors, +PD032 or +CHIR) even in the high dose of LIF. Scale Bar 200μm.

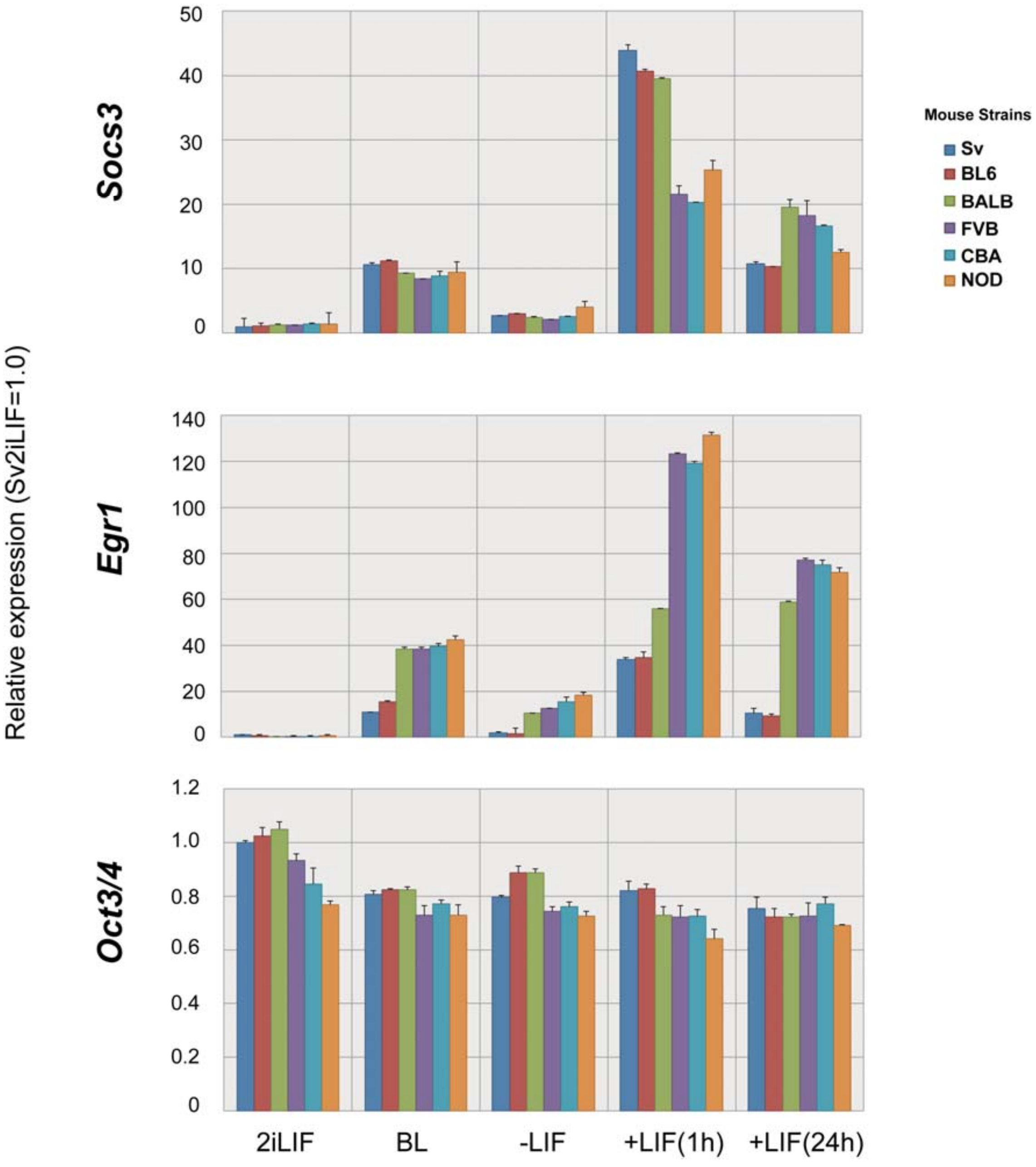

Supplemental Figure S3. LIF-responsive in serum-free culture condition.

First the ES cells were plated, cultured for 24 hours in 2iLIF medium. As the second step, the medium was changed to serum-free N2B27 medium supplemented with BMP and LIF and cultured for another 24 hours (BL). As the third step, LIF was

washed out with N2B27 followed by the culture in cells in N2B27 medium supplemented with BMP without LIF for 24 hours (B-LIF). Finally, 10^3 units/ml of of LIF was added to the medium and harvested cells at the time points indicated (+LIF 1h and 24h) for RNA preparation. Q-PCR analysis were performed as Figure 2C for Socs3 and Egr1 and the relative expression units were presented with standard error (set the expression levels of 129Sv ES cells in 2iLIF culture at 1.0).

Table S1.

Click here to Download Table S1
